# Identifying Agricultural Frontiers for Modeling Global Cropland Expansion

**DOI:** 10.1016/j.oneear.2020.09.006

**Published:** 2020-10-23

**Authors:** Felix Eigenbrod, Michael Beckmann, Sebastian Dunnett, Laura Graham, Robert A. Holland, Patrick Meyfroidt, Ralf Seppelt, Xiao-Peng Song, Rebecca Spake, Tomáš Václavík, Peter H. Verburg

**Affiliations:** 1 School of Geography and Environmental Science, University of Southampton, Southampton, UK; 2 Department of Computational Landscape Ecology, UFZ—Helmholtz Centre for Environmental Research, 04318 Leipzig, Germany; 3 Earth and Life Institute, UCLouvain, 1348 Louvain-la-Neuve, Belgium; 4 Fonds de la Recherche Scientifique (F.R.S.- FNRS), 1000 Brussels, Belgium; 5 iDiv—German Centre for Integrative Biodiversity Research, 04103 Leipzig, Germany; 6 Institute of Geoscience & Geography, Martin-Luther-University Halle-Wittenberg, 06099 Halle (Saale), Germany; 7 Department of Geosciences, Texas Tech University, Lubbock, TX 79409, USA; 8 Department of Ecology and Environmental Sciences, Faculty of Science, Palacký University Olomouc, 78371 Olomouc, Czech Republic; 9 Global Change Research Institute of the Czech Academy of Sciences, 60300 Brno, Czech Republic; 10 Institute for Environmental Studies, VU University Amsterdam, de Boelelaan 1087, 1081HV Amsterdam, the Netherlands; 11 Swiss Federal Institute for Forest, Snow and Landscape Research, Birmensdorf, Switzerland

**Keywords:** climate change, land use change, integrated assessment models, sustainability, agriculture, deforestation, cropland expansion, frontier dynamics, positive deviance analysis

## Abstract

The increasing expansion of cropland is major driver of global carbon emissions and biodiversity loss. However, predicting plausible future global distributions of croplands remains challenging. Here, we show that, in general, existing global data aligned with classical economic theories of expansion explain the current (1992) global extent of cropland reasonably well, but not recent expansion (1992–2015). Deviations from models of cropland extent in 1992 (“frontierness”) can be used to improve global models of recent expansion, most likely as these deviations are a proxy for cropland expansion under frontier conditions where classical economic theories of expansion are less applicable. Frontierness is insensitive to the land cover dataset used and is particularly effective in improving models that include mosaic land cover classes and the largely smallholder-driven frontier expansion occurring in such areas. Our findings have important implications as the frontierness approach offers a straightforward way to improve global land use change models.

## Highlights

• The current (1992) global extent of cropland can be explained with existing data
• Recent expansion (1992–2015) of cropland is not well explained with existing data
• Deviations from the 1992 model of extent improve models of cropland expansion

## Science for Society

Cropland area is increasing globally to satisfy the growing population and consumption rates. Cropland expansion often comes at the expense of forests, which are critical for conserving biodiversity and mitigating against climate change. Therefore, it is essential to know where cropland expansion is likely to occur in the future in order to design policies that prevent expansion in areas that are most likely to conflict with forest conservation. However, predicting where expansion is most likely is difficult as few data are available on key predictors related to governance. Here, we devise a novel, two-stage method for predicting expansion of cropland. First, available data are used to model where cropland existed in 1992. We then use maps that show where the first model fails to explain cropland in 1992 to help predict expansion of cropland between 1992 and 2015. We show that this approach is an improvement over simply using existing data to predict recent expansion of cropland.

## Graphical Abstract

**Figure:**
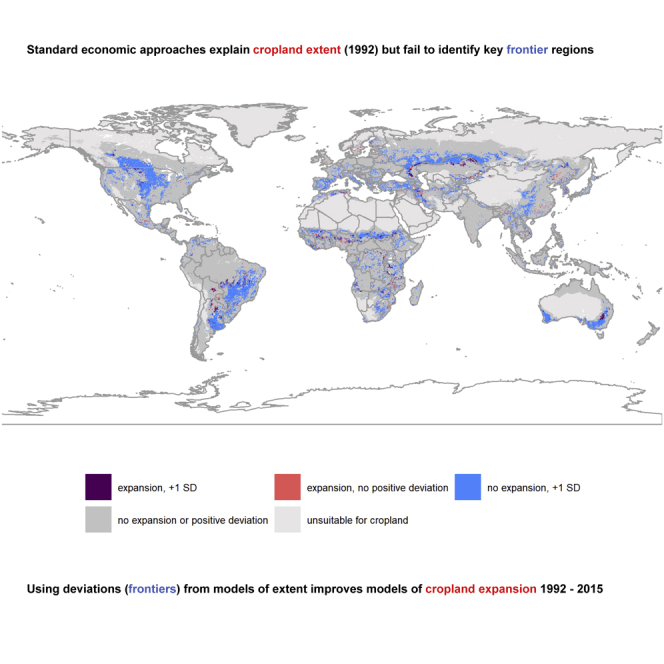

## Teaser

Predicting where cropland is likely to expand globally has important implications for climate change and biodiversity. However, doing so is challenging because of a lack of data on key drivers of current expansion in so-called frontier areas (e.g., governance). Here, we show that using deviations from a model of cropland extent in 1992 built on all available data improves models of recent (1992–2015) cropland expansion over and above using the best available existing data.

## Introduction

The increasing appropriation of land by humans is a major driver of global environmental change.^1^^,^^2^ Cropland expansion is a particular issue, given the projected increase in demand for agricultural products driven by changing dietary patterns of a growing population, increased demand for land-based climate solutions,^3^ and the potential threat such expansion has for high-biodiversity tropical areas.^4^, ^5^, ^6^ While intensification of agriculture on existing agricultural lands can reduce the need for expansion into uncultivated areas,^7^^,^^8^ the rate of global yield gains per area is decreasing,^9^ and the rate of agricultural expansion has been increasing since 2000, mostly in the tropics.^10^ However, much uncertainty exists about how much future expansion of cropland is likely to occur globally,^11^ and where this will occur.^12^, ^13^, ^14^ This creates uncertainty in cropland expansion projections, which in turn has major implications for global models of land-based climate change mitigation options, and of biodiversity loss.^11^^,^^12^

A key challenge to forecasting cropland expansion is the uncertainty as to whether the identity and magnitude of predictors of cropland expansion are consistent across space and time. While such consistency may be true in consolidated agricultural regions, such as Europe,^15^ this is unlikely to be true globally, because frontier expansion of agriculture occurs under very different circumstances than in consolidated agricultural regions.^16^^,^^17^ Frontiers are regions with rapid land use expansion and an imbalance between abundant land and natural resources and a relative lack of capital or labor to exploit these resources.^16^^,^^18^ In frontier areas, case studies and theory suggest that agricultural expansion occurs under imperfect market conditions where agency of key actors, rent-capture behaviors, and non-equilibrium dynamics dominate.^18^ Large-scale actors operating in commodity frontiers have the agency to modify the very conditions (such as accessibility, infrastructures, or policies) that influence their own cropland expansion.^18^ By contrast, in smallholder-driven frontiers, coexistence between market-oriented and subsistence production and imperfect market integration are such that decisions may differ from the simple economic optimum captured in coarse-scale global approaches.^19^^,^^20^ Finally, the globalized nature of trade in land-based products, such as agricultural commodities and timber^21^ may lead to amplification of regional disparities in land use trajectories, further increasing global differences between past and current predictors of cropland expansion.

Frontier dynamics remain poorly represented in global land use models^13^ as even the most recent models are largely based on classical land rent theories under equilibrium conditions.^20^^,^^22^ Moreover, systematic tests of the effects of these assumptions on the accuracy of the predictions of global land use models remain limited,^23^ and the spatial and temporal differences in the identity and effects of putative predictors of agricultural expansion have yet to be tested at the global scale. This lack of testing represents a key research gap for predicting future land cover changes.^24^

Until recently, a major barrier to assessing whether predictors of land use change are stable or changing over time has been the lack of finely resolved, global data on land use change. Such data are now available, at least for recent changes in forest cover^25^ and land cover (1992–2015).^26^ A recent global analysis suggested that forest loss between 2000 and 2015 was driven both by commercial agriculture and shifting (subsistence) agriculture,^27^ but did not directly assess how this relates to recent agricultural expansion, nor whether predictors of forest loss are stationary over time. Therefore, the need remains for an improved global, spatially resolved understanding of which socio-ecological factors are the most important predictors of recent agricultural expansion,^28^ and if and how these differ from the factors that explain the current global extent of cropland.

Here, we address this challenge in two ways. First, we use logistic regression models to compare how well globally available, independent putative predictors of agriculture explain global cropland *extent* (in 1992) and recent cropland *expansion* (1992–2015). This provides a first global test of the degree to which the consistency and magnitude of predictors of cropland remain consistent over time. Globally available data enable tests of classical theories of cropland expansion but not of theories of recent expansion in agricultural frontiers^18^^,^^20^ (Table 1), so our *a priori* hypothesis was that these data would better predict extent than expansion of cropland, as the latter is likely mostly occurring in frontier regions.^20^

**Table 1:** Independent Global Predictor Variables and Their Theoretical Justification for Cropland Expansion

Variable	Theoretical Justification
Bioclimatic Suitability^29^	Ricardian land rent theory^30^
Steepness^29^	Ricardian land rent theory^30^
Access (distance to markets)^31^	von Thünen's location theory^32^
Bioclimatic Suitability ∗ Access OR Steepness ∗ Access	interactions between land rent theory and location theory (i.e., steep land is more valuable near markets)
Population Density (1990)^33^	induced intensification^34^
GDP (1992)^35^	market demands for agriculture^20^
Population Density ∗ Bioclimatic Suitability OR Steepness OR Access OR GDP	interactions between induced intensification, land rent theory and between induced intensification and location theory or market versus subsistence demands for agriculture
GDP ∗ Access OR Steepness OR Biophysical constraints	interactions between subsistence and market demands on agriculture^20^

These predictor variables were used to explain cropland extent in 1992 and expansion between 1992 and 2015 (Model 1), and construct the Null Model of current cropland extent. The ∗ represents interaction terms between variables. Full details of the justification for these variables are in the Experimental Procedures.

Second, we applied positive deviance analysis^36^ to the extent of cropland in 1992 to develop and test the utility of a new proxy for recent agricultural expansion frontiers (which we refer to hereon as “*frontierness*”). Positive deviance (or “bright spot”) analysis has been used in fields, including human development,^36^ conservation,^37^ and ecosystem services,^38^ to identify locations where the outcome variable exceeds (or falls below, cf. “dark spots”) expectations derived from a “*Null Model*.” The Null Model should be clearly linked to hypotheses about known factors.^38^ Here, the Null Model corresponds to the classical theories of agricultural expansion that can be quantified globally with available data (Table 1) (Experimental Procedures). Positive deviations from the Null Model are therefore locations with cropland in 1992 *not* predicted by classical theories of agricultural expansion, and as such potentially indicative of pre-1992 cropland expansion frontiers. We then tested if these deviations from the Null Model of extent in 1992—frontierness—can explain expansion of cropland from 1992 to 2015. The hypothesis we test here is that pre-1992 frontiers are correlated with short- to medium-term post-1992 cropland expansion frontiers.

We show that, in general, existing global data, which are aligned with classical theories of agricultural expansion, explain the current global extent of cropland reasonably well, but do not explain most recent cropland expansion well. However, our proxy for frontiers (frontierness) generally provides significant improvements to models of 1992–2015 cropland expansion. Our findings have important implications for global land use change models, as they show both that the dependence of these models of classical theories of agricultural expansion is problematic, but also that they can be improved using the frontierness approach we demonstrate here.

## Results

### Standard Theories Explain Extent Better Than Expansion

**Figure 1:**
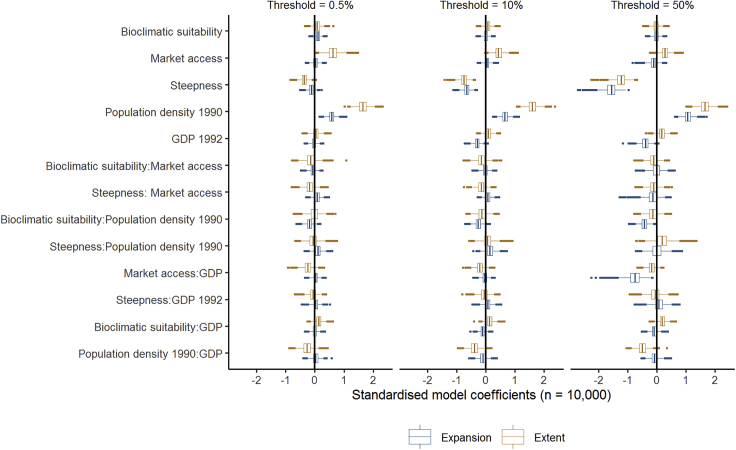
Explanatory Power of Standard Theories for Extent and Expansion Comparison of the explanatory power of global datasets aligned with classical theories of cropland expansion (Table 1) for the global extent of cropland (1992) and recent expansion of cropland (2015) (Model 1; Experimental Procedures). Boxplots of the coefficient estimate for all predictor variables in Model 1 from 10,000 individual models. Each model represents a balanced sample (each with 500 cropland and 500 non-cropland pixels). Models were run for 3 different binary thresholds (0.5%, 10%, and 50%) of minimum levels of cropland per 5′ × 5′ pixel are shown (Experimental Procedures). Interactions between predictors are shown using “:”; e.g., Population Density 1990:GDP.

In general, independent global predictor variables aligned with classical theories of cropland expansion (Table 1; Figure 1 and Model 1; Experimental Procedures) explained the 1992 extent of cropland globally well, but not recent expansion (1992–2015). However, the choice of threshold used to categorize land into non-cropland and cropland (to perform logistic regressions) had a major effect on the expansion analyses, although not the extent analyses (Figure 1). Expansion events were characterized as extreme (that is, very high prevalence of within-pixel expansion) (>50% of a 5′ × 5′ pixel, subsequently expansion_50%_), major (>10% of a 5′ × 5′ pixel, subsequently expansion_10%_), or minor (>0.5% of a 5′ × 5′ pixel, subsequently expansion_0.5%_). Extreme within-pixel expansion events are relatively predictable using existing global datasets (average McFaddden pseudo-*R*^2^ expansion_50%_ = 0.28), but these events are globally very rare (only 0.45% of expansion events at the 0.5% threshold are also above the 50% threshold; Figure S1 in Supplemental Information). However, most major and minor recent expansion is poorly predicted (pseudo-*R*^2^ expansion_10%_ = 0.12; expansion_0.5%_ = 0.08) using these existing data. By contrast, results for extent were relatively consistent, irrespective of the threshold chosen (average McFadden pseudo-*R*^2^: extent_0.5%_ = 0.32, extent_10%_ = 0.30, extent_50%_ = 0.33).

The magnitude and even direction of the effects of some predictors varied across models of cropland extent and recent expansion. The effect of GDP (a proxy for economic activity) was positive for the extent models, but negative for the expansion models. At a 50% threshold, “access” (distance to markets) had a positive relationship with cropland for the extent model, but a negative effect on expansion. The considerably higher explanatory power of the 50% threshold expansion models relative to the lower thresholds appears to be due to the much stronger negative effect of “steepness” (percentage cover of steep slopes), and to a lesser degree the positive effect of population density for this threshold relative to expansion at other thresholds. Differences between thresholds for the extent models were less pronounced, but steepness also had a higher explanatory power for extent of agriculture in 1992 for the 50% threshold than the other two thresholds (Figure 1). See Figure S2 in the Supplemental Information for global distributions of extent and expansion of cropland at all three thresholds.

### Expansion Aligns with Positive Deviance

**Figure 2:**
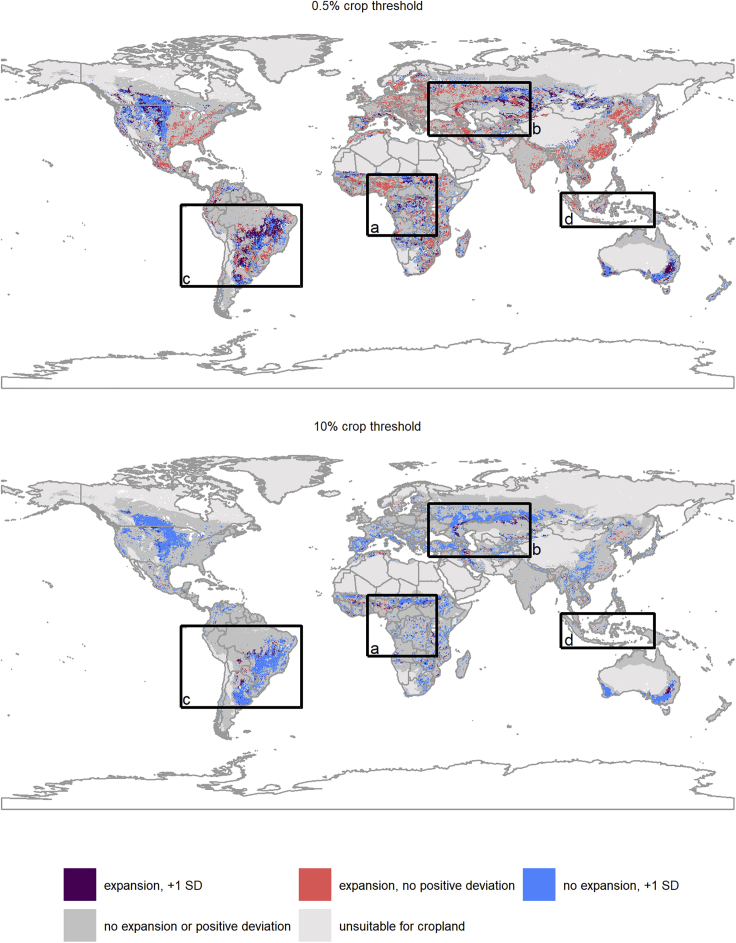
Global Overlaps of Expansion and Frontierness The overlap of recent cropland expansion (1992–2015) and high frontierness (>1 positive deviations) from global 1992 Null Models of cropland extent at the 0.5% (top panel) and 10% (bottom panel) threshold of minimum levels of cropland per 5′ × 5′ pixel (Experimental Procedures). Results using both thresholds are shown to highlight differences in the distribution of expansion frontiers and how these overlap with the >1 SD positive deviations. Global results are shown; close-ups from key cropland expansion frontiers (boxes) are shown in Figure 3. Results for the 50% expansion threshold are not shown here as they are too rare to visualize at the global scale. See Figure S2 in the Supplemental Information for global distributions of extent and expansion of cropland at the >0.5%, >10%, and >50% binary expansion thresholds.

A disproportionate amount of recent expansion of cropland (0.5% or 10% threshold) occurs in areas that have at least 1 SD or more cropland in 1992 (positive deviations) than would be predicted by the predictors of classical theory within the Null Model (Table 2; Figure 1). Such areas of high positive deviations correspond to high values of frontierness (all deviations from the 1992 Null Model; Null Model coefficients in Table S1). Recent expansion at the 0.5% threshold is extremely common (21% of bioclimatically suitable areas), including within regions already dominated by agriculture (Europe, eastern USA, China), as well as within frontier regions in South America, sub-Saharan Africa, and the former USSR. Overlaps with positive deviations of 1 or more SD is high within the latter areas—with the exception of Indonesia—but not the former. Major expansion (10% threshold) is much less common, and concentrated in frontier regions, where it shows a high degree of overlap with positive deviations from the Null Model (Figure 2). Conversely, very rare, extreme expansion (50% threshold) occurs in areas with less cropland in 1992 (negative deviations) than would be predicted by classical theory (Table 2).

**Table 2:** Proportional Overlap of Expansion of Cropland between 1992 and 2015 and Deviations from the Global Null Model of Cropland Extent in 1992 (Experimental Procedures; Null Model)

	Expansion of Cropland 1992–2015		
	0.5% Crop	10% Crop	50% Crop
Positive Deviance 2SD	1.35	3.05	0.03
Positive Deviance 1SD	1.53	2.58	0.03
Negative Deviance 2SD	0.41	0.39	2.41
Negative Deviance 1SD	0.34	0.43	3.40

The predictor variables in the Null Model are the same as in Model 1 (Table 1), but the Null Model uses all data globally; Model 1 uses balanced samples of 500 presences and absences (Experimental Procedures). “Positive Deviance” and “Negative Deviance” refer to 2 or 1 or more positive (or negative) standard deviations (2SD and 1SD) from the Null Model. Ratios >1 indicate overrepresentation; ratios <1 indicate underrepresentation (more or less overlap of cropland and a given deviation threshold than would be expected if both are equally common across the land area they cover^39^).

### Frontierness Improves Models of Expansion

Inclusion of frontierness as a predictor (Model 3) increased the predictive power of the 0.5% and 10% threshold 1992–2015 expansion models by a pseudo-*R*^2^ of 0.08 over models using existing predictors (Model 2), but did not improve the predictive power of the 50% threshold expansion model (Table 3).

**Table 3:** Average Predictive Power (McFadden Pseudo-R^2^) of Models with and without Frontierness for Recent (1992–2015) Expansion of Cropland

Expansion Threshold	Existing Predictors (Model 2)	Frontierness + Existing Predictors (Model 3)
0.5% cropland	0.12	0.20
10% cropland	0.23	0.31
50% cropland	0.41	0.41

Model 2 includes all independent predictors of expansion aligned with classical theories of expansion (that is all predictors in Model 1 and the Null Model; outlined in Table 1), as well as the percentage of cropland in 1992 as a predictor, and its interaction with Bioclimatic Suitability, Access, Population Density in 1990 and GDP in 1992. The inclusion of existing cropland is a standard practice in global models predicting land use change. Model 3 includes all terms in Model 2, as well as frontierness, and the interaction of frontierness and cropland in 1992 (Experimental Procedures). Results are averages of 1000 balanced sub-samples of expansion for each model (Experimental Procedures). Full model results are given in the Supplemental Information for Model 2 (Table S3) and Model 3 (Table S4).

## Discussion

A key challenge for predicting future land use change globally is understanding the degree to which the predictors of such changes are stationary in space and time.^12^^,^^28^ Here, we show that, while classical theories of cropland expansion explain the current (1992) extent of cropland reasonably well, this is generally not the case for recent cropland expansion (1992–2015). We also show that using deviations from a Null Model of cropland extent in 1992 (frontierness) improves our ability to predict recent expansion. Both results have important implications for global land use models.

At present, approaches to modeling gridded global land use and land cover change generally use existing cropland and available land as initial inputs for prediction, in addition to measures of regional disaggregated demand for agricultural products.^12^ Some gridded models also assume expansion after accounting for constraints (e.g., protected area coverage, bioclimatic limits of cropland, other existing land cover types) and endogenous suitability, such as soil type and aridity.^24^^,^^40^ Our findings show that this approach is unlikely to predict future agricultural expansion well, most likely because most current cropland expansion is driven by multiple processes and actors that are not fully captured by existing global datasets.^17^^,^^18^ Key commercial crops do disproportionately overlap with cropland expansion at all thresholds (Table S8); however, such expansion can be driven both by large commercial land holders^41^ and smallholders.^42^ More generally, the disproportionate overlap of expansion at the 0.5% and 10% threshold with both large and small fields (Table S8) supports existing work showing that frontier expansion is driven both by commercial and subsistence activities.^16^, ^17^, ^18^^,^^43^

**Figure 3:**
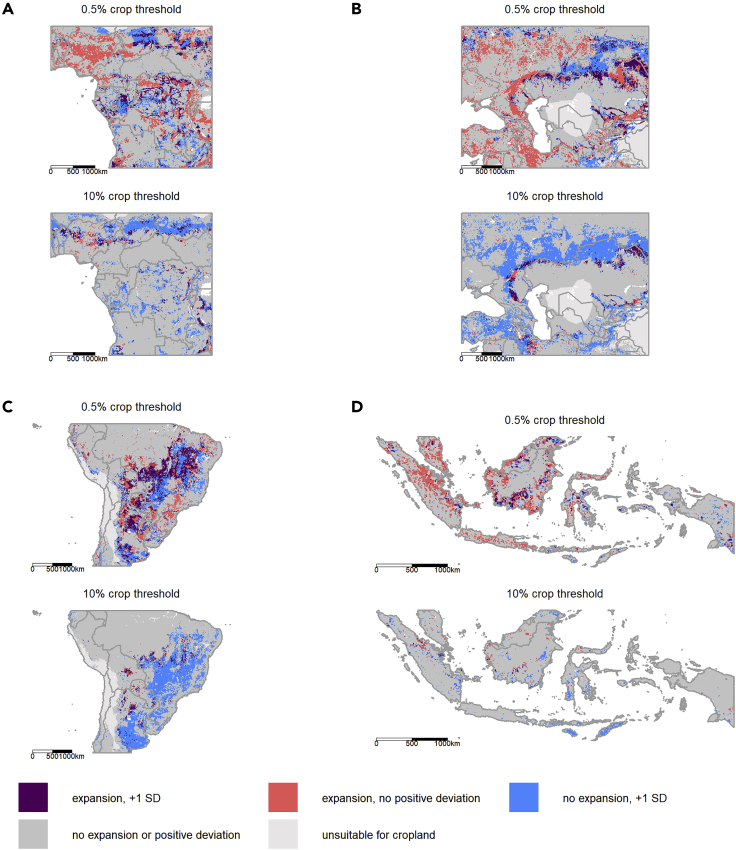
Overlaps of Expansion and Frontierness for Key Expansion Frontiers The overlap of recent cropland expansion (1992–2015) and large (>1 SD) positive deviations from global 1992 Null Models of cropland extent at the 0.5% (top panel) and 10% (bottom panel) threshold (Experimental Procedures) for key cropland expansion frontiers. The letters correspond to the locations of the regions on the global map (Figure 2).

Our proxy for agricultural expansion frontiers—frontierness—added explanatory power to models of agricultural expansion, except for the most extreme, and very rare, expansion events (>50% threshold). The spatial pattern of our frontierness indicator appears to align with recent cropland expansion in areas recognized as key global cropland expansion frontiers in South America (Cerrado and Chaco ecoregions^41^), as well as forest frontiers in West Africa, and a large arc of cropland expansion in the steppes of Southern Russia and Kazakhstan—some of these the result of recultivation of land abandoned at the collapse of the Soviet Union^44^, ^45^, ^46^ (Figure 3). This is likely because the post-1992 expansion frontiers occur largely in the same places as recent, pre-1992 expansion frontiers. Indeed, *post-hoc* analyses showed proportionally similar or higher overlap with areas of high positive deviation from the Null Models of cropland extent for agriculture-driven deforestation in the period 1982–1992 as compared with 1992–2015, as measured using an independent global dataset^47^ (Supplemental Information; Supplemental Experimental Procedures and Table S9). This suggests that at present there is at least some medium-term (∼30 years) stationarity in the bulk of global cropland expansion frontiers. This is likely because expansion frontiers build on agglomeration economies, which require some time to appear as cropland expands in a region, and thus cropland expansion disproportionately occurs near places where some expansion has already taken place.^20^^,^^48^ As such, at the 5′ × 5′ arc min resolution (roughly 10 × 10 km at the equator), pixels can remain “expansion” pixels over a fairly long time period, as gradually more of their area is converted to cropland. Interestingly, *post-hoc* overlap analyses (Table S2) demonstrated that areas with more cropland than predicted (high positive deviations from the Null Models of cropland extent in 1992) disproportionately overlapped with the largest fields, as measured in 2017.^49^ This might reflect the recent shifts to large-scale, commercial agriculture in frontier areas, as observed in South America,^41^ and that recent expansion is predominantly associated with large-scale actors more generally. This *post-hoc* analysis also showed no tendency of positive deviations toward less developed countries (as measured by political stability or human development index [HDI]), and only a weak positive effect of favorable soil conditions, again illustrating the difficulty in *a priori* identification of expansion frontiers using existing datasets. Overlaps of these variables with areas with high negative deviations from the predicted extent of cropland in 1992 (so less cropland than expected) did appear to be disproportionately located in areas with major soil constraints; this perhaps explains the overlaps with small field sizes in such areas. As the frontierness proxy is all deviations from the Null Model (positive and negative), it suggests that negative values of the proxy flag areas where local conditions (e.g., soil constraints) limit cropland expansion that is otherwise predictable from existing global datasets.

As current frontiers can be identified using our positive deviance frontierness approach for the most current available land cover data, this means we can use the approach to predict medium-term future frontier areas with currently available data within existing predictive models of land use change. Frontierness could therefore be used to help close a major research gap—building spatially dissagregated transfer functions within gridded economic models.^28^ For example, frontierness could be an additional input into global, country-level economic models, enabling the modeling of within-country spatial heterogeneity in land supply elasticities, building on recent work where market access was used within such models.^50^ Moreover, the same positive deviance approach that underpins frontierness could potentially be used for improving models of other types of land use or land use intensity change. Of course, if the nature of expansion changes—for example, to more rapid, large-scale expansion in frontier areas, as appears to be the case in South America^41^—the predictive power of frontierness may decrease in the future. However, even where regions are changing, frontierness could still be useful, as a way of helping to identify outlier regions, where additional case studies and ground-based analyses could help understand why they deviate from expectations and thereby enrich land use theories. For example, the relative *lack* of overlap of frontierness with recent major (>10%) expansion in Indonesia (Figure 3) could be partially linked to the 2011 moratorium on new land concessions in Indonesia, which slowed deforestation since 2012.^51^ To encourage further use of our proxy, we provide global geospatial files of frontierness—calculated for both 1992 (as used in this paper) and for 2015 (latest data) as outlined in the Data and Code Availability in the Resource Availability section.

Counterintuitively, very rare, extreme expansion (50% threshold) can be relatively well explained by existing predictor variables. *Post-hoc* overlap analysis showed that such expansion has a disproportionate overlap with modeled distributions of soy and very large fields (Supplemental Information; Table S8). Large fields are often associated with large farms, which in turn relate to farm income.^43^^,^^52^^,^^53^ As such, observed extreme expansion fits with the commodity and neoliberal resource frontier theories of agricultural expansion. Here, rapid land use expansion for large-scale agriculture occurs in resource frontiers where there is an abundance of land on which structural factors, such as new agricultural technologies, infrastructure, and rising producer prices, facilitate large agricultural rents, but the expansion strongly depends on the ability of powerful actors to capture or influence these rents through large-scale, capital-intensive agriculture (e.g., le Polain de Waroux and colleagues^18^^,^^54^). This might include influencing the location of new roads or infrastructures, such as ports, or even building these themselves, influencing the spatial pattern and implementation of land use policies, such as areas and practices targeted for agricultural subsidies or where land uses are restricted.^18^ This might explain why these rare expansion events exhibit a negative, rather than positive response to distance to market and population density. Market access may also be acting as a proxy for some predictors of commodity frontiers as it is inversely correlated with poverty and low land costs.^55^ Our ability to explain these very rare, extreme expansion events reasonably well with global data may be also partly be due to a relatively small number of 5ʹ × 5′ pixels globally that are (1) flat enough for intensive mechanized agriculture (steepness is a key predictor of extreme expansion); (2) where some cropland already exists (agriculture in 1992 is a key predictor as well), but (3) there is still enough space for the ∼25 km^2^ (at the equator) of new cropland this threshold entails. The relatively high predictability of rare, extreme cropland expansion events is an important finding, as while very rare, such events represent the extreme points of wider, industrial-scale expansion frontiers. Such areas (e.g., the South American resource frontiers, which are increasingly characterized by large field sizes^41^) are known to have major social and ecological consequences.^54^^,^^56^

Importantly, our frontierness approach is robust to how cropland is defined, the choice and thematic resolution of the inputted cropland map, as well as the length of the time series considered. Results of including mosaic land cover classes (hereafter “mosaic”) were similar to the main analyses for the extent analyses (Table S5). The explanatory power of existing predictors (Model 2) was lower in the mosaic analyses for the 10% and 50% thresholds for the base expansion models (0.16 versus 23 and 0.30 versus 0.41, respectively). However, importantly, for the models that included frontierness (Model 3), the explanatory power of the mosaic models was identical to the main results (Table S6). These results suggest that existing predictors capture mosaic expansion poorly, likely as such mosaic expansion represents a smallholder-driven expansion frontier. Fortunately, our frontierness proxy is effective in explaining such mosaic expansion frontiers when the Null Model includes mosaic land cover classes in estimates of cropland. The choice of end date for the expansion analyses was unimportant; end dates of 2013 and 2015 yielded almost identical explanatory power of all expansion models (Table S6). Finally, results of extent analyses using either the European Space Agency Climate Change Initiative (ESA CCI) data (main results) and MODIS data^57^ for 2001–2015 also differed negligibly, likely as both datasets accurately represent the majority of cropland at the global scale50 (Supplemental Experimental Procedures; Figures S3 and S4; Table S7). However, the explanatory power of models using MODIS data was much higher than the CCI data for the 2001–2015 expansion analyses; indeed, expansion is better predicted than extent based on the MODIS data (Table S7). Critically, adding frontierness still greatly improved the explanatory power (pseudo-*R*^2^) of the MODIS expansion models (from 0.35 to 0.53 and 0.42 to 0.57 for 0.5% and 10% cropland, respectively) (Table S7), again illustrating the robustness of our frontierness approach to varying definitions of cropland. The relative predictability of expansion as measured by MODIS is perhaps because underrepresentation of cropland by the MODIS data in 2001 (Figure S3) meant more of the measured expansion 2001–2015 occurred within relatively predictable consolidated agricultural regions as opposed to frontier regions (Figure S4).

As with any global analyses, our work here is subject to a number of caveats. Firstly, we were unable to address adequately in the extent analysis the issue of endogeneity of human population density, GDP, and cropland extent. Population growth is a known driver of cropland expansion,^58^ but people are drawn to existing agricultural areas, as reflected by the establishment of many of the world's great cities within areas with rich agricultural lands. As population density is measured in 1990, and cropland and GDP in 1992, it is impossible to ascertain statistically which ones are causal. The issue of endogeneity also affects the “market access” layer, as this is based on city populations and human populations in 2000.^31^ Unfortunately, these issues of endogeneity cannot be addressed as no spatially resolved global primary datasets of human population exist before 1990; as such the extent analyses should be viewed with more caution than the expansion analyses, which do not suffer from this problem. Secondly, as gridded global time series for key potential drivers of expansion (e.g., population) do not exist before 1990, we were unable to test if past trajectories of change of population (or GDP) can improve the predictive power of models of cropland expansion and reduce the utility of frontierness. Changes in population density, HDI, and GDP between 1990 and 2015 (*post-hoc* overlap analyses; see Supplemental Experimental Procedures and Table S8) were not associated with cropland expansion at the 0.5% and 10% threshold. However, it is possible that longer-term, and lagged changes in socio-economic variables could be important predictors of future expansion; testing this should be a priority as such data become available.

Accurate predictions of cropland expansion are critical in understanding both issues of food security and climate change,^59^ but also how these in turn affect biodiversity.^6^^,^^60^ This study shows that, in general, recent cropland expansion cannot be explained well with currently available data. Our study also highlights the value of using residual deviations from a model of current extent of cropland (our frontierness proxy) as a new way of improving the predictive power of models of cropland expansion over and above what is possible with globally available predictor variables. This is likely because these residuals, potentially representing expansion frontiers, are not currently captured in global models,^13^ and is particularly true for models that include mosaic land cover classes. Taken together, our results both show the limits of current data and approaches for predicting cropland globally, and offer some novel and promising ways forward for such work.

## Experimental Procedures

### Resource Availability

#### Lead Contact

Further questions about the analysis should be directed to and will be fulfilled by the Lead Contact, Felix Eigenbrod (f.eigenbrod@soton.ac.uk).

#### Materials Availability

This study did not generate new unique materials*.*

#### Data and Code Availability

The code used for all analyses generated during this study, as well as .tif files of frontierness for 2001 and 2015, are freely available on Mendeley Data: https://doi.org/10.17632/rtgdj2jmk6.1^61^

### Methods

#### Cropland Data

We derived global data on cropland in 1992 and 2015 using the ESA's CCI 2015 dataset, which provides harmonized land cover for all years globally between 1992 and 2015.^26^ While the ESA CCI land cover is available at 300 × 300 m resolution, our analyses were conducted at 5′ × 5′ arc min resolution (percentage of available land that is cropland), as this is the finest common resolution at which the predictor variables for the models of extent and expansion used here are available. To minimize the effects of errors in the ESA CCI 2015 data,^62^ we excluded pixels classified as mosaic cropland (classes 30 and 40) and only consider those pixels classified as 100% cropland (classes 10,11, 12,20) as cropland in this analysis. We calculated the increase in cropland between 1992 and 2015 at the 300 × 300 m resolution simply by identifying pixels classified as cropland as outlined above in 2015, but not classified as cropland in 1992. We then calculated the percentage cropland cover (for 1992) and change in cropland cover (1992–2015) per 5′ × 5′ arc min pixel (approximately 10 × 10 km at the equator), using the ESA CCI 150 × 150 m resolution water mask. We then converted percent cropland per 5′ × 5′ pixel to a binary variable, as with other recent work^27^^,^^63^ as the extremely zero-inflated nature of the 1992–2015 expansion data precludes statistical analysis of the raw percentage cropland data. As the threshold used to binarize the cropland variable greatly affects the number of recent cropland expansion pixels globally (see Supplemental Information; Figure S1), we used three different thresholds both for extent and for expansion: >0.5% cropland, >10% cropland, and >50% cropland. Pixels classified as having undergone expansion had an increase in cropland corresponding at least to these thresholds between 1992 and 2015.

As all global cropland maps have considerable error associated with them,^62^ we also ran a series of sensitivity analyses to assess whether our findings were robust to how cropland was measured. Firstly, we re-ran the extent and expansion analyses, using a reclassified ESA CCI map that reclassed the mosaic land cover classes (classes 30 and 40) as cropland. This is because excluding these classes—as is the case in the main analysis as outlined above—could underestimate the total amount (1992) and expansion (1992–2015) of cropland. This could have disproportionately affected smallholder systems, and hence smallholder expansion-driven frontiers. Secondly, we checked the robustness of our results to the choice of end date used for the expansion analysis, by re-running all expansion analyses using an end date of 2013 rather than 2015. Finally, as land cover products vary enormously in their cropland estimates,^62^ we also re-ran all extent and expansion models for both the ESA CCI dataset (used in the main analysis), and the MODIS 6 land cover product^57^ over the period 2001–2015 for which time series are available for both datasets. These datasets represent opposite extremes in estimates of cropland globally, with MODIS generally underestimating cropland, and ESA CCI data prone to overestimating cropland.^62^ Full details of all sensitivity tests are in the Supplemental Experimental Procedures.

#### Socio-economic and Biophysical Explanatory Datasets

We assembled global spatial datasets independent of land cover relevant for cropland extent and expansion that are available at 5′ × 5′ resolution or finer (Table 1). This is because using data modeled using land cover to explain a land cover type (cropland) would lead to circularity in our inference.^64^ These are as follows: (1) *Bioclimatic suitability*. This is the summed, standardized agroclimatic potential for low-input, rainfed agriculture of the main crops globally—wheat, soy, maize, and rice, as well as oil palm.^29^ The latter is included due to its recent disproportionate role in driving recent agricultural expansion in sub-Saharan Africa.^42^ Note this excludes edaphic constraints, as these are partially based on land cover. (2) *Steepness.* This is the percentage of each pixel with a slope of >15%, which is the limit to mechanized agriculture.^29^ (3) *Access.* This is the distance to markets.^31^ (4) *Population density* (in 1990).^33^ (5) *GDP*. GDP in 1992.^35^ We do not consider dynamic variables (e.g., change in population density or GDP) in any models as (1) no relevant gridded global datasets exist before 1990; and (2) using dynamic data between 1990 and 2015 to predict expansion between 1990 and 2015 would be circular.

We then built statistical models that linked the above variables to *a priori* theories of cropland expansion (Table 1). Bioclimatic suitability and steepness relates to Ricardian^30^ land rent theory, which states that “rent” (underlying value) of land for agriculture is related to its biophysical characteristics relative to other land under use. Distance to markets captures von Thünen's location theory^32^ that the highest land rent is near markets, which makes it most likely to be converted to agriculture. We captured the interactions between the biophysical (Ricardian) and location-based determinants of land rent for agriculture using interaction terms (Equation 1). These simple models do not encompass other key determinants of cropland expansion in land rent theory—high agricultural prices and low alternative employment opportunities,^65^ as such factors are not available globally in spatially disaggregated form. We also included human population density, which relates to Boserup's (1965) theory^34^ arguing that expansion of agriculture occurs to satisfy subsistence needs until land becomes scarce, after which intensification takes over. Of course, population expansion can also occur after cropland is established. Unfortunately, high-resolution gridded population data do not exist earlier than 1990, so we were unable to account for this problem of potential endogeneity. Grid cells with high population density should—all other things being equal (modeled via the biophysical constraints, access to markets and interactions between population, access, GDP in 1992, and biophysical constraints; Equation 1)—be dominated by agriculture. By including interactions between economic activity (as measured by GDP) and access, steepness, as well as agroclimatic potential (Equation 1), we incorporated hypotheses concerning potential interactions between subsistence and market demands for agriculture.^20^ However, these variables do not take into account the technological, institutional variables that also determine demand per unit area—(induced intensification theory), or quality of governance, inequality in access to land, and other factors that influence the heterogeneity in the agent's ability to materialize potential land rents, all of which are important in determining modern resource frontiers.^18^^,^^20^ Again, this is due to data constraints.

#### Explaining Extent and Expansion with Standard Theories

Like-to-like comparisons of the relative importance of different predictors explaining recent agricultural expansion (1992–2015) and cropland extent in 1992 requires a sub-sampling approach.^15^^,^^66^ This is because locations of expansion events are comparatively extremely rare to locations where expansions did not occur. Sub-sampling approaches enable balanced sampling, where an equal number of “presences” (changes) and “absences” are randomly sampled many times from across the study area, and the average coefficients are used.^15^^,^^66^^,^^67^ This approach also reduces the spatial autocorrelation.^68^ Therefore, we randomly selected 500 presence and 500 absence squares globally 10,000 times for all three cropland thresholds (0.5%, 10%, or 50%, or more expansion or extent in cropland) for both extent and expansion models, and then ran a logistic regression models for each sub-sample.

In each case, we used the same global logistic regression model (Model 1) to enable like-to-like comparisons. This model includes variables that relate to the major existing theories of agricultural expansion.^20^^,^^65^

Cropland [balances sample of extent in 1992 OR expansion between 1992 and 2015] ∼ X1 *(Model 1)*

where X1 = Bioclimatic Suitability + Access + Steepness + Population Density + GDP + Bioclimatic Suitability ∗ Access + Steepness ∗Access + Bioclimatic Suitability ∗ Population Density + Steepness ∗ Population Density + GDP ∗ Access + GDP ∗ Steepness + GDP ∗ Bioclimatic Suitability + GDP ∗ Population Density

All statistical analyses were limited to areas considered minimally bioclimatically suitable (values >0) for rainfed cultivation of at least one of wheat, soy, maize, rice, or oil palm by the FAO.^29^ This excludes deserts, high mountains, and the Artic. It also excludes some irrigated croplands in deserts (e.g., Nile valley; Indus valley); this is because spatial data on such irrigated areas are only available using datasets partially based on cropland data,^69^ which would introduce circularity into the analysis.

In addition, models of expansion were limited to bioclimatically suitable pixels that had sufficient space for agricultural expansion at the relevant threshold. For example, at the 10% threshold, at least 10% of the pixel had to be available for expansion. To limit expansion to realistic pixels,^24^ the only land within a pixel considered for expansion was areas that were not (1) cropland, urban areas, snow/ice, water, or barren land/rock in in 1992; and (2) covered by protected areas (IUCN classes I to IV),^70^ in 1992.

All variables were centered and standardized globally across suitable pixels, and re-centered for each sub-sample (as recommended for models with interaction terms).^71^ We then calculated the average coefficient, 95% confidence interval, and average McFadden pseudo-*R*^2^ across all 10,000 models in each instance.

### Positive Deviance Analysis

Positive deviance (or bright spot) analysis identifies locations where the outcome variable exceeds expectations (areas with high positive residuals) derived from a Null Model. Here, the global null model (Null Model) corresponds to all theories of agricultural expansion that can be quantified globally with available data (Table 1); that is, the same predicator variables used for the extent to expansion comparison (Model 1). As existing data do not capture recent frontier expansion well (Experimental Procedures; Q1), positive deviations (areas with more cropland than predicted in 1992) may represent cropland frontier expansion events (frontierness). The Null Models are run for all three cropland thresholds (>0.5%, >10%, and >50%) and use all bioclimatically suitable pixels (approximately 1.3 million) because positive deviance analysis cannot be carried via the sub-sampling approach used for Model 1. Our use of positive deviance analysis is somewhat different from other socio-ecological approaches in that our primary interest is not in explaining positive and negative deviations in the 1992 cropland per se, but rather if our putative proxy for expansion frontiers (frontierness; deviations from the null 1992 model) can predict post-1992 cropland expansion events.

Cropland extend in 1992 (globally) ∼ X1 *(Null Model)*

where X1 = Bioclimatic Suitability + Access + Steepness + Population Density + GDP + Bioclimatic Suitability ∗ Access + Steepness ∗Access + Bioclimatic Suitability ∗ Population Density + Steepness ∗ Population Density + GDP ∗ Access + GDP ∗ Steepness + GDP ∗ Bioclimatic Suitability + GDP ∗ Population Density

#### Does Expansion Align with Positive Deviance?

We quantified the proportional overlap of cropland expansion between 1992 and 2015 at different expansion thresholds (0.5%, 10%, and 50% cropland) with areas covered by positive and negative deviations at least 1 and 2 SD (respectively) from the global 1992 extent model, following Eigenbrod and colleagues.^39^ Deviation thresholds that had more of their summed total value overlapping with cropland expansion at a given threshold than expected for the area of the cropland have values >1; deviation thresholds that are under-represented by a given cropland expansion dataset have values below 1. In every case, we used the same cropland threshold (0.5%, 10%, and 50%) for both the Null Model and the extent of cropland expansion.

To better understand what the deviations from the 1992 Null Model (frontierness) represents, we also did *post-hoc* overlap analyses of frontierness and spatial variables associated with frontier expansion. This approach has previously been used for negative deviance (bright spot) analyses in environmental studies.^37^^,^^38^ The variables we considered include: (1) the HDI in 1992^35^; (2) political stability (a country-level indicator from the Global Governance Index [only political stability was chosen, *sensu*,^72^ as all six Global Governance indices are highly correlated]); (3) the distribution of the largest and smallest quartiles of field size,^49^ and (4) soil constraints on plant growth.^29^ The soil constraint layer comprised binary pixels (5′ × 5′ min resolution); those classified as “1” have none of the seven constraints on plant growth in the FAO dataset classified as higher than “moderate”; areas classified as “0” have at least one constraint of “severe” or above. None of these variables were suitable for the regression models used in the main analysis, being either too coarse in resolution (political stability), partially derived from land cover (field size, soil constraints), or highly correlated with an existing variable (HDI is highly correlated with GDP), but all link to existing theories of frontier expansion.^20^

#### Does Frontierness Improve Models of Expansion?

We used logistic regression to formally test whether frontierness (deviations from the null 1992 cropland model) represents a useful proxy for expansion frontiers between 1992 and 2015. Our approach is analogous to that of a recent study that uses deviations from a null (biophysical only) model as a new variable for quantifying the impacts of technological advances on banana production globally.^73^ The use of residuals (or deviations) as a predictors is generally considered poor practice if applied to the same response model;^74^ however, this is not the case here; cropland in 1992 is independent of expansion of cropland between 1992 and 2015.

To test if frontierness provides additional explanatory power over existing datasets, we compared two different models for each of the three cropland thresholds (0.5%, 10%, and 50%). The first—Model 2—includes the terms (X1) as in Model 1 (the extent to expansion comparison) and the Null Model—as are all globally available independent predictors of expansion—as well as the percentage of cropland in 1992. We include the latter as a predictor, as current extent of cropland is known to be a very good predictor of future expansion, and is therefore incorporated into most land use change models.^12^ As such, Model 2 represents the current state of the art in terms of statistical modeling of global cropland expansion. The third model—Model 3—includes all variables in Model 2, as well as frontierness. We include interaction terms between cropland in 1992 and all other independent predictors as all could potentially moderate the influence of existing cropland on expansion in both Model 2 and Model 3. We also include the interaction term between cropland in 1992 and frontierness in Model 3, as areas with existing cropland in frontier areas could plausibly have higher likelihood of expansion as other areas. We do not include interactions between frontierness and the other independent predictors, as we do not have *a priori* justification for doing so. The models are as follows:

Cropland [expansion between 1992 and 2015] ∼ X1 + X2 *(Model 2)*

Cropland [expansion between 1992 and 2015] ∼ X1 + X2 + X3 *(Model 3)*

where X1 = Bioclimatic Suitability + Access + Steepness + Population Density + GDP + Bioclimatic Suitability ∗ Access + Steepness ∗Access + Bioclimatic Suitability ∗ Population Density + Steepness ∗ Population Density + GDP ∗ Access + GDP ∗ Steepness + GDP ∗ Bioclimatic Suitability + GDP ∗ Population Density.Where X2 = Percentage Cropland 1992 + Percentage Cropland 1992 ∗ Bioclimatic Suitability + Percentage Cropland 1992 ∗Access + Percentage Cropland 1992 ∗ Steepness + Population Cropland 1992 ∗ Population Density + Percentage Cropland 1992 ∗ GDP 1192.Where X3 = Frontierness + Frontierness ∗ Percentage Cropland 1992

All analyses were conducted using R 3.6,^75^ using a variety of packages.

## Supplemental Information

Document S1. Supplemental Experimental Procedures, Figures S1–S4, and Tables S1–S9

Document S2. Article plus Supplemental Information
