# Supplementary material for: Identifying Agricultural Frontiers for Modeling Global Cropland Expansion
Source: One Earth. 2020 Oct 23;3(4):504–14. doi: 10.1016/j.oneear.2020.09.006 (PMC7608111; doi:10.1016/j.oneear.2020.09.006)
Supplement: Document S1. Supplemental Experimental Procedures, Figures S1–S4, and Tables S1–S9 [file mmc1.pdf]

One Earth, Volume *3*

Supplemental Information

Identifying Agricultural Frontiers

for Modeling Global Cropland Expansion

Felix Eigenbrod, Michael Beckmann, Sebastian Dunnett, Laura Graham, Robert A. Holland, Patrick Meyfroidt, Ralf Seppelt, Xiao-Peng Song, Rebecca Spake, Tomáš Václavík, and Peter H. Verburg

Supplementary Information

-Supplementary Figures
-Supplementary Experimental Procedures
-Supplementary Tables
-Supplementary References

Supplementary Figures

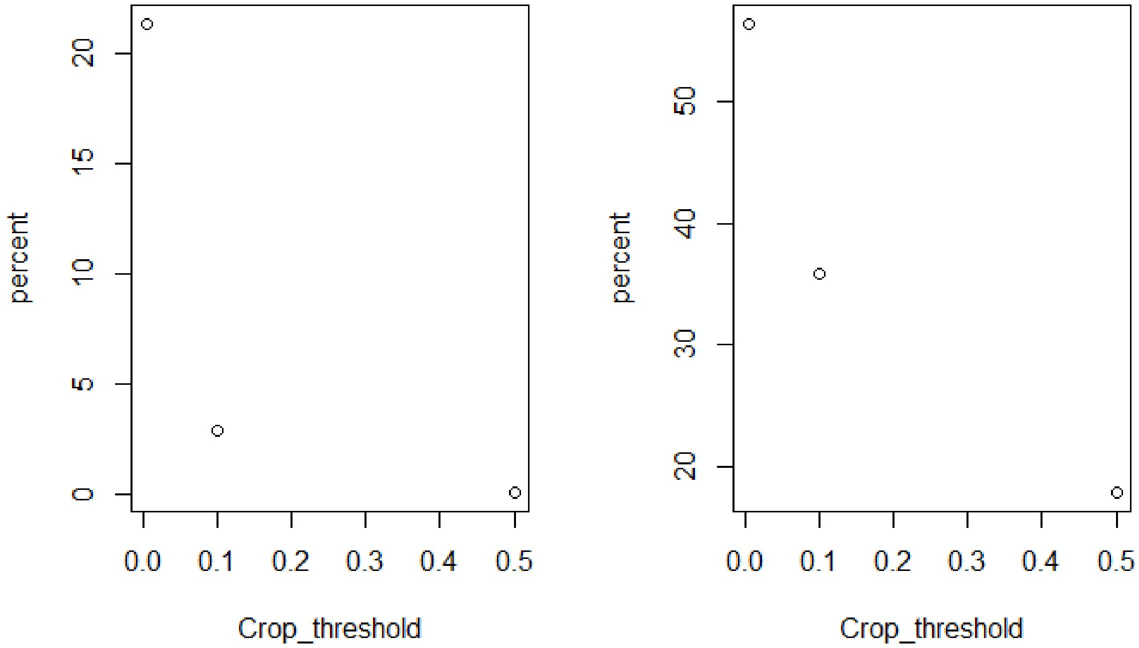

Fig S1 Relationship between the percentage of bioclimatically suitable land area for cropland and the threshold of the percentage of cropland per 5' x 5' arc minute grid cell (Crop_threshold) for recent expansion (1992 to 2015; left) and cropland extent in 1992; right). The value near 0 is for a threshold of 0.005 (0.5%) cropland per grid cell. In total, 0.45% of expansion at the 0.5% threshold is also above the 50% cropland threshold; 13.6% of expansion at the 0.5% threshold is also above the 10% cropland threshold. Cropland is based on ESA CCI data and excludes mosaic land cove classes (Methods).

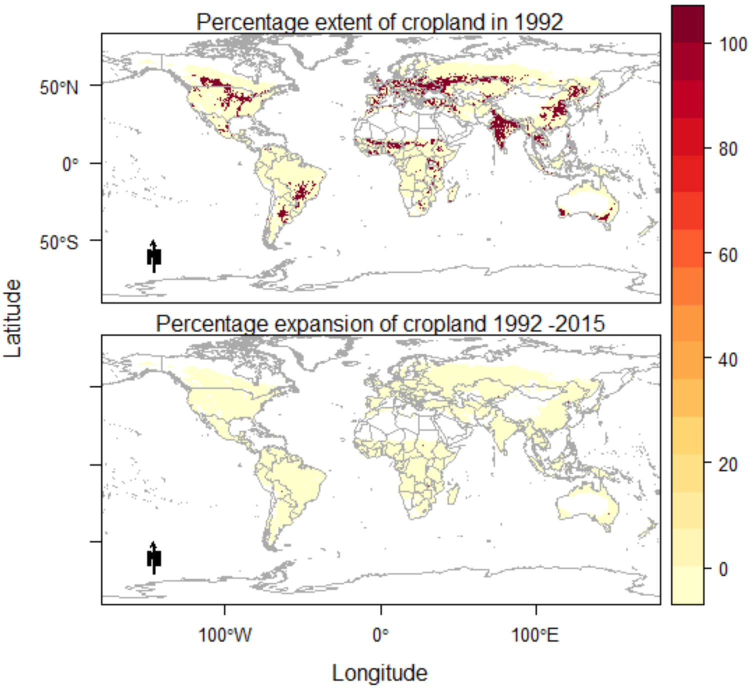
Percentage extent of cropland in 1992
Percentage expansion of cropland 1992 -2015
50°N
0°
50°S
Latitude
100°W
0°
100°E
Longitude
100
80
60
40
20
0

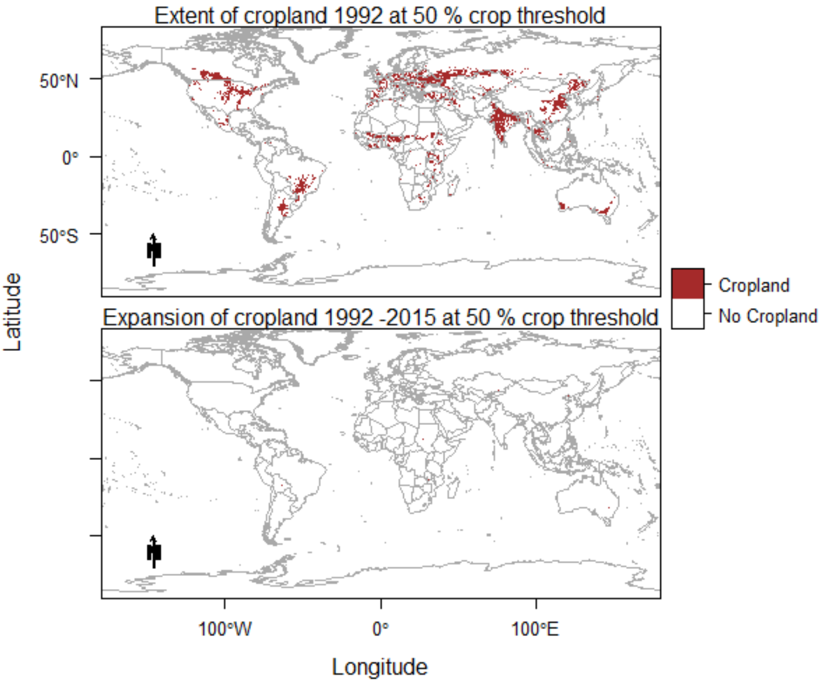
Extent of cropland 1992 at 50 % crop threshold
Expansion of cropland 1992 -2015 at 50 % crop threshold
50°N
0°
50°S
Latitude
100°W
0°
100°E
Longitude
Cropland
No Cropland

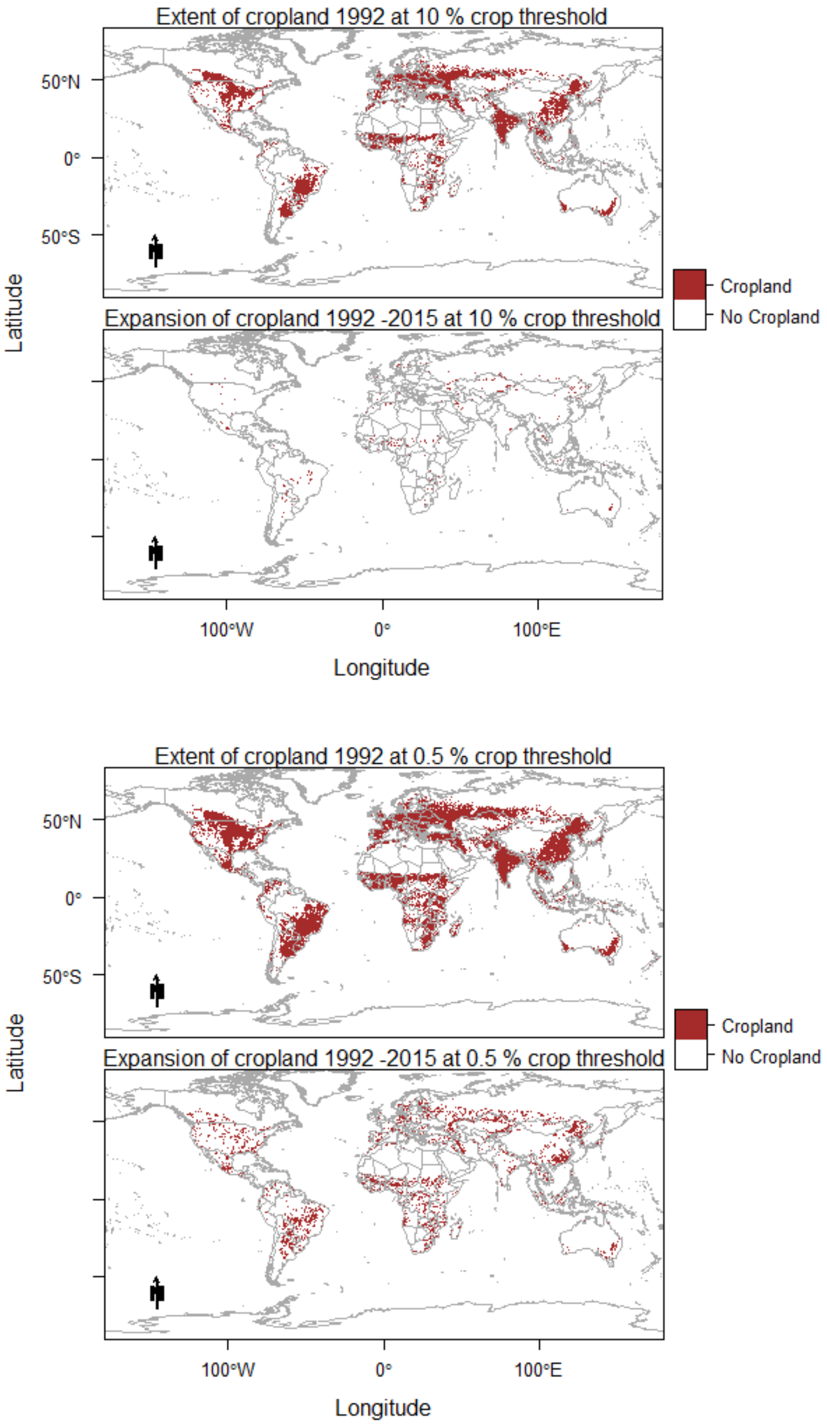

Fig. S2 Distribution of the global extent (in 1992) and recent expansion (1992-2015) of cropland in terms of percentage per 5' x 5' pixel, and at different binary thresholds of cropland per pixel (>0.5%; >10% and >50%). In all cases, only cropland within the bioclimatic envelope (Methods) for rain-fed agriculture is shown, as this was data used in the analysis. Cropland is based on ESA CCI data and excludes mosaic land cove classes (Methods). Note that expansion events at the 50% threshold are extremely rare (~1300 pixels globally), making it difficult to distinguish them in a global map.

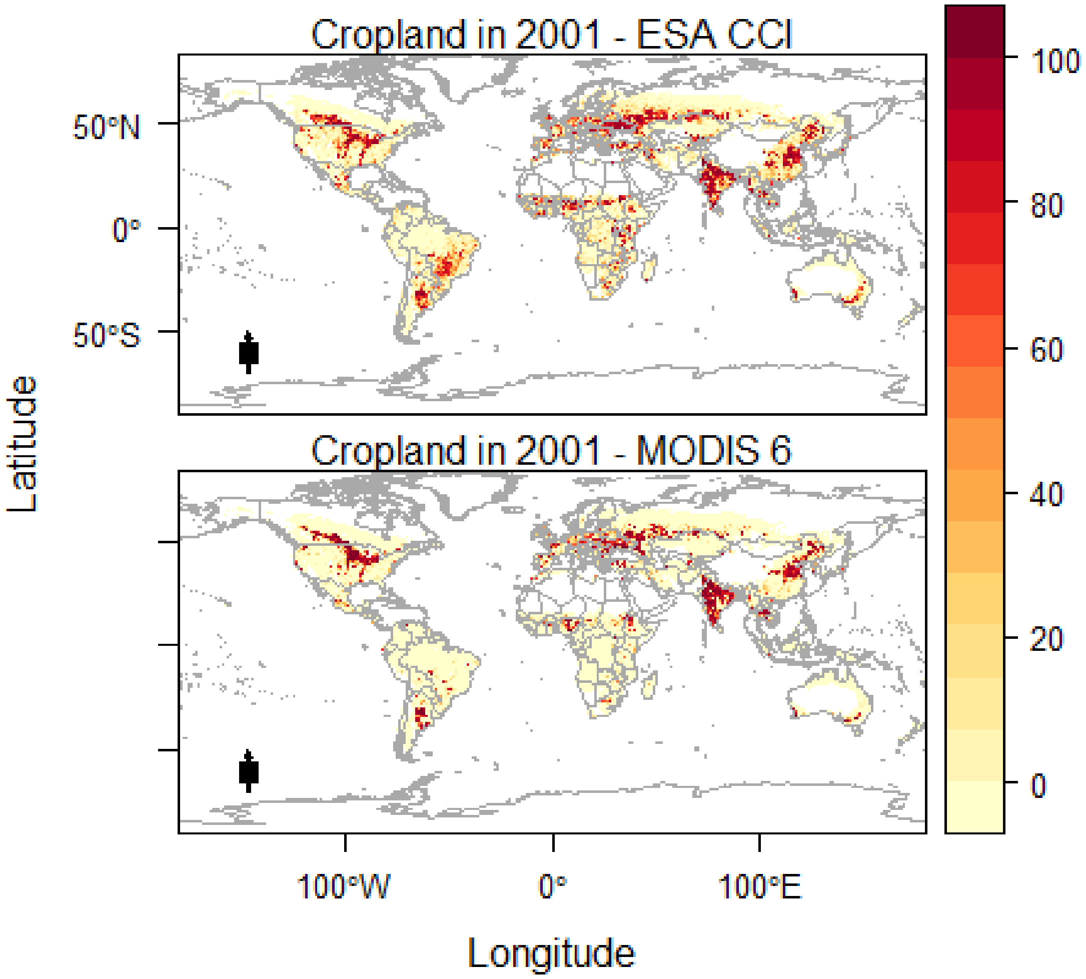

Fig S3 – Distributions of percentage cropland per 5' x 5' pixel in 2001 as measured by the ESA CCI (top) and MODIS 6 (bottom) data. In both cases, mosaic land cover classes are excluded in the calculations of the distribution of cropland. See Supplementary Methods for details on dataset creation.

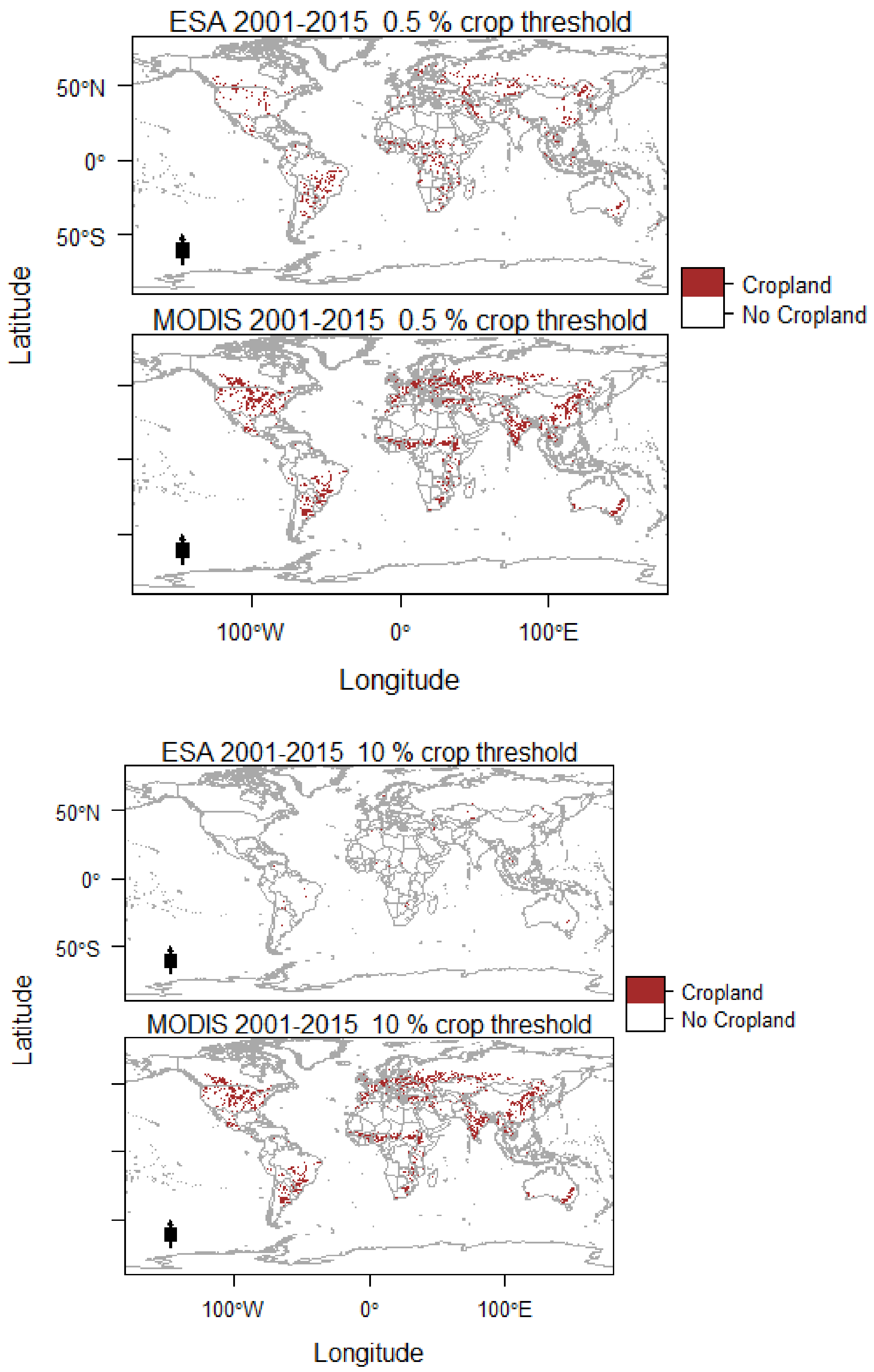

Fig S4: Distribution of expansion (2001-2015) of cropland as measured using the ESA CCI and MODIS 6 land cover products at different binary thresholds of cropland per 5’ x 5’ pixel (>0.5% and >10%). In all cases, only cropland within the bioclimatic envelope (Methods) for rain-fed agriculture is shown, as this was data used in the analysis. In both cases, mosaic land cover classes are excluded in the calculations of the distribution of cropland. See Supplementary Methods for detailed methods.

Supplementary Experimental Procedures

Post-hoc proportional overlap of expansion and other socio-economic variables.

We undertook post-hoc analyses of the proportional overlaps of recent cropland expansion and a number of additional socio-economic variables. This enabled us to better understand what is driving this expansion (Table S6). These were: 1) change in HDI and 2) change in GDP between 1992 and 2015; [1]; 3) change in human population between 1990 and 2015[2], 4) the largest and 5) smallest quartiles of field size)[3]; 6) percentage irrigated area ([4]; locations where deforestation between 2003 and 2015 was classified as primarily caused by 7) commercial agriculture and 8) subsistence agriculture [5]; and distributions of 9) oil palm and 10) soy[6]. These analyses were carried out post-hoc, and only via overlap analyses, as none of these datasets are suitable for predictive modelling of cropland expansion. This because socio-economic change data (so change in HDI, GDP and populations) is only relevant for explaining changes that have already occurred, not prediction into the future, while the field size, irrigated area, deforestation, and oil palm and soybean datasets all include cropland data within them, and hence would introduce circularity in statistical testing. Moreover, the cropland data used in the above datasets comes from multiple years between 1992 and 2015. As such, the results of these overlaps should be viewed with considerable caution. However, comparing the overlaps between the different threshold of cropland expansion (0.5%, 10% and 50%) does provide some additional insights of whether expansion at different thresholds is driven mainly by corporate actors (associated with very large fields dominated by cash crops such as soy and oil palm) or smallholders[7,8]. Variables that had more of their summed total value overlapping with cropland expansion at a given threshold than expected for the area of the cropland have values > 1; Variables that are under-represented by a given cropland expansion dataset have values below 1. This is the same method as used for cropland expansion and deviations from the Null Model (Methods). Results are shown in Table S8.

Post-hoc proportional overlap of forest to short vegetation transitions between 1982 and 1992 and 1992 and 2015 and areas of high positive deviance from the null model

To better understand what the frontierness proxy (deviations from the global Null Model of cropland extent in 1992; Methods) is measuring, we conducted additional post-hoc analyses using a recent long-term time-series of vegetation changes (1982-2016) measured using AVHRR data[9]. Specifically, we looked at the proportional overlap of transitions from forests to short vegetation between 1982 and 1992 and areas of high deviation (+1 and +2 SD, respectively) using the proportional overlap method outlined above. Our hypothesis is that if these deviations represent recent expansion frontiers, these areas of deforestation would be disproportionately overrepresented by these areas of high deviation. However, the AVHRR data is not directly comparable to the ESA CCI dataset we use for recent land use change. The AVHRR-based forest-to-short vegetation transition represents deforestation for both cropland and pasture expansion[9]. This can lead to major discrepancies between the approaches. For example, post-2000, new croplands in tropical South America were mostly converted from pasture [10]. Indeed, the global Spearman correlation between non-zero shifts from forest to short vegetation and the ESA CCI derived percentage increase in cropland at the 5' resolution is only 0.18. We therefore also quantified the proportional overlap of the AVHRR data between 1992 and 2015, thereby enabling us to make a like-to-like comparison of how well areas of high deviation from the 1992 Null model overlap with deforestation frontiers before and after 1992.

As breaking the 1982 to 2016 time series used in Song et al into two separate time slices reduces the sample size for per-pixel trend analyses, we ran the 1982-1992 and 1992-2015 analyses at 0.08°, rather than 0.05° in the original analysis. Specifically, we first resampled the annual tree cover (TC) and short vegetation (SV) layers from 0.05° to 0.04° using bilinear resampling and aggregated them from 0.04° to 0.08° – taking the mean value of each 2 x 2 window. We tested non-parametric trends with the aggregated data, separately for TC and SV, following [9]. Then, a TC-to-SV transition was identified such that TC showed a statistically significant decreasing trend ($p<0.05$) and SV showed a statistically significant increasing trend ($p<0.05$). We computed the Theil-Sen slopes for TC change and SV change, and took the smaller of the two as the annualized TC-to-SV change. We then converted it to total percentage change over the length of the specific time period. This was carried out separately for 1982-1992 and 1992-2015. Results are shown in Table S9.

Sensitivity testing of results to the assumptions of the ESA CCI crop map

In the main analysis, we excluding mosaic land cover classes. This could have disproportionately affected smallholder systems, and hence smallholder expansion driven frontiers. We therefore re-ran our analyses to include cropland represented by mosaic land cover classes, as well as the 100% cropland (10, 11, and 12) land cover classes. The two mosaic land cover classes in the ESA CCI dataset are 30 (>50% cropland) and 40 (<50% cropland). In absence of additional data, we assumed class 30 was 75% cropland, and class 40 25% cropland. Results are shown in Table S5 ('extent') and Table S6 ('expansion').

As land cover products vary enormously in their estimates of cropland[11], we also re-ran all 'extent' and 'expansion' models for both the ESA CCI dataset (used in the main analysis), and the MODIS 6 land cover product[12] over the period (2001-2015) for which time series are available for both datasets. We only considered 'pure' cropland classes (100% cropland; classes, 10, 11, and 20 for ESA CCI; class 12 for MODIS 6 to align with the main analysis (distributions shown in Fig S3). We also followed the same method for calculating cropland change as for the main analysis (distributions shown in Fig S4). Specifically, we calculated the increase in cropland between 2001 and 2015 at the 300 x 300 m resolution (for the ESA CCI data) or 500 x 500 m resolution (MODIS 6 data) by

identifying pixels classified as cropland as outlined above in 2015, but not classified as cropland in 2001. We then calculated the percentage cropland cover (for 2001) and change in cropland cover (2001 to 2015) per 5' x 5' arcminute pixel (approximately 10 km x 10 km at the equator), using the ESA CCI 150 x 150 m resolution water mask (resampled for the MODIS data). We then calculated the land available for crops in 2001 using the same methods as before (though run at 500 x 500 m resolution for the MODIS data), but used world protected area database from 2001 instead of 1992[13] to exclude protected areas within these calculations. We then re-ran all 'extent' (Model 1) and 'expansion' (Model 2 and Model 3) analyses, using the same equations by updated key predictors to align as closely as possible to 2001 (as opposed to 1992 in the base analysis). Specifically, instead of using population density in 1990[2], we used population density in 2000[14]; GDP in 2001 instead of 1992[1]. Frontierness in the 'expansion' analysis was always based on the deviations from the corresponding 'extent' analysis (e.g. frontierness in the 'expansion' analysis using MODIS data for 2001-2015 was based on the 'extent' analysis based on MODIS data for 2001).

Finally, we checked to see if our results were robust to potential noise in the specific endpoint we used (2015). Specifically, we re-ran our main 'expansion' analysis for the years 1992-2013, rather than 1992-2015 (Table S5).

Creation of geospatial files of frontierness for 1992 and 2015

We provide global 5' x 5' resolution geospatial files (.tif) of frontierness for 1992 and 2015, based on the main analysis (ESA CCI data; mosaic land cover classes excluded). Values for both files are standardized between 0 and 1, exclude areas that are not bioclimatically suitable (Methods), and are in geographic projection. The 1992 file is the standardized deviations from the global Null Model, as described in the Methods. Note Population Density is from 1990:

$$\begin{aligned}\text{Cropland} \in 1992(\text{globally})\ &\text{Bioclimaticsuitability} + \text{Access} + \text{Steepness} + \text{Populationdensity} \\ &+ \text{GDP1992} + \text{Bioclimaticsuitability} * \text{Access} + \text{Steepness} * \text{Access} \\ &+ \text{Bioclimaticsuitability} * \text{Populationdensity} + \text{Steepness} * \text{Populationdensity} \\ &+ \text{GPD1992} * \text{Acccess} + \text{GDP1992} * \text{Steeepness} + \text{GDP1992} \\ &* \text{Bioclimaticsuitability} + \text{GDP1992} * \text{Populationdensity}\end{aligned}$$

The 2015 file is identical in structure, but with the 2015 cropland data as the response, and updated datasets where these are available (GDP for 2015 and Population Density for 2015). The 2015 GDP data comes from [1] (the same as the 1992 GDP data), while the Population Density data for 2015 comes from SEDAC v4 [14] (as opposed to SEDAC v3 for the 1990 data[2]).

Supplementary Tables

Table S1 – Model coefficients and McFadden pseudo-R^2 of the Null Model (Methods) of global cropland extent in 1992. The deviations from this model form the basis of the frontierness proxy.

Mean ± SE	> 0.5% cropland in 1992	> 10% cropland in 1992	> 50% cropland in 1992
Intercept	0.495±0.003	0.003±-0.894	-0.894±0.003
Bioclimatic suitability	0.06±0.003	0.003±0.112	0.112±0.003
Market access	0.622±0.005	0.005±0.375	0.375±0.003
Steepness	-0.355±0.003	0.003±-0.733	-0.733±0.004
Population Density 1990	1.597±0.004	0.004±1.571	1.571±0.005
GDP 1992	0.011±0.003	0.003±0.14	0.14±0.003
Bioclimatic suitability * Market Access	-0.171±0.004	0.004±-0.124	-0.124±0.003
Steepness * Market Access	-0.179±0.004	0.004±-0.149	-0.149±0.003
Bioclimatic suitability * Population Density 1990	-0.02±0.004	0.004±-0.137	-0.137±0.004
Steepness * Population Density 1990	-0.069±0.004	0.004±0.044	0.044±0.005
Market Access * GDP 1992	-0.239±0.004	0.004±-0.174	-0.174±0.003
Steepness * GDP1992	-0.053±0.003	0.003±-0.028	-0.028±0.004
Bioclimatic suitability * GDP1992	0.138±0.003	0.003±0.107	0.107±0.003
Population Density 1990 * GDP 1992	-0.266±0.004	0.004±-0.423	-0.423±0.004
McFadden Pseudo-R^2	0.307±0.307	0.307±0.288	0.288±0.288

Table S2 – Proportional overlap of expansion of variables potentially associated with frontier conditions, and deviations from the global 'null' model of cropland extent in 1992 (Experimental Procedures; Null Model). The predictor variables in the Null Model are the same as in Model 1 (Table 1), but the Null Model uses all data globally; Model 1 uses balanced samples of 500 presences and absences (Experimental Procedures). 'Positive Deviance' and 'Negative Deviance' refer to 2 or 1 or more positive (or negative) standard deviations (2SD and 1SD) from the Null Model. Ratios > 1 indicate over-representation; ratios < 1 indicate under-representation (more or less overlap of given variable and a given deviation threshold than would be expected if both are equally common across the land area they cover [15].

Threshold	Variable	Positive Deviance 2 SD	Positive Deviance 1 SD	Negative Deviance 2 SD	Negative Deviance 1 SD
0.5%	Largest quartile of field sizes	**1.82**	**2.32**	0.38	0.38
0.5%	Smallest quartile of field sizes	0.05	0.40	**1.70**	0.83
0.5%	HDI in 1992	**1.22**	**1.05**	**1.03**	0.88
0.5%	Political stability	**1.18**	**1.05**	0.98	0.91
0.5%	Soil constraints	**1.25**	**1.10**	0.17	0.07
10%	Largest quartile of field sizes	**3.16**	**2.56**	0.52	0.62
10%	Smallest quartile of field sizes	0.26	0.99	**2.14**	**1.55**
10%	HDI in 1992	**1.16**	**1.04**	0.94	0.95
10%	Political stability	**1.14**	**1.02**	0.94	0.96
10%	Soil constraints	**1.12**	**1.26**	**1.05**	0.97
50%	Largest quartile of field sizes	**3.53**	**2.76**	0.58	0.71
50%	Smallest quartile of field sizes	0.73	**1.15**	**2.83**	**2.03**
50%	HDI in 1992	**1.15**	**1.08**	0.87	0.98
50%	Political stability	**1.11**	**1.03**	0.89	0.98
50%	Soil constraints	**1.36**	**1.47**	**1.17**	**1.06**

Table S3 – Average model coefficients and McFadden pseudo-R^2 (± SD) of 10,000 balanced samples of expansion (1992-2015) of Model 2 (‘existing predictors’); Methods.

	**> 0.5% cropland**	**> 10% cropland**	**> 50% cropland**
**Mean ± SD**			
X-Intercept	0.33 ± 0.08	0.23 ± 0.09	0.02 ± 0.12
****			
Bioclimatic suitability	0.10 ± 0.08	-0.02 ± 0.09	-0.12 ± 0.12
****			
Market access	0.13 ± 0.10	0.08 ± 0.10	-0.08 ± 0.13
****			
Steepness	0.06 ± 0.12	-0.34 ± 0.14	-1.42 ± 0.25
****			
Population Density 1990	0.38 ± 0.13	0.09 ± 0.15	0.31 ± 0.18
****			
Percent Agriculture 1992	0.46 ± 0.13	1.09 ± 0.15	1.37 ± 0.17
****			
GDP 1992	-0.13 ± 0.09	-0.42 ± 0.11	-0.53 ± 0.15
****			
Bioclimatic suitability * Market Access	-0.03 ± 0.10	0.02 ± 0.11	0.10 ± 0.17
****			
Steepness * Market Access	-0.04 ± 0.10	0.02 ± 0.10	-0.11 ± 0.20
****			
Bioclimatic suitability * Population Density 1990	-0.11 ± 0.12	-0.09 ± 0.13	-0.26 ± 0.15
****			
Steepness * Population Density 1990	-0.03 ± 0.13	0.06 ± 0.15	-0.06 ± 0.25
****			
Market Access * Percent Agriculture 1992	-0.13 ± 0.09	-0.16 ± 0.11	-0.37 ± 0.14
****			
Steepnes * Percent Agriculture 1992	0.37 ± 0.16	0.30 ± 0.16	0.05 ± 0.20
****			
Bioclimatic suitability * Percent Agriculture 1992	-0.08 ± 0.10	-0.16 ± 0.13	-0.21 ± 0.18
****			
Population Density 1992 * Percent Agriculture 1992	-0.50 ± 0.13	-0.50 ± 0.16	-0.24 ± 0.19
****			
Market Access * GDP 1992	-0.02 ± 0.10	-0.04 ± 0.10	-0.71 ± 0.23
****			
Steepness * GDP1992	0.09 ± 0.10	0.11 ± 0.12	0.02 ± 0.23
****			
Bioclimatic suitability * GDP1992	-0.05 ± 0.09	-0.20 ± 0.10	-0.25 ± 0.12
****			
Population Density 1990 * GDP1992	0.02 ± 0.13	0.05 ± 0.13	0.17 ± 0.16
****			
McFadden Pseudo R2	0.12 ± 0.02	0.23 ± 0.02	0.41 ± 0.03

Table S4 – Average model coefficients and McFadden pseudo-R^2 (± SD) of 10,000 balanced samples of expansion (1992-2015) of Model 3 (‘existing predictors + frontierness’); Methods.

	**> 0.5% cropland**	**> 10% cropland**	**> 50% cropland**
**Mean ± SD**			
X-Intercept	0.19 ± 0.25	2.10 ± 0.37	-0.19 ± 0.18
****			
Bioclimatic suitability	0.12 ± 0.08	-0.06 ± 0.10	-0.06 ± 0.13
****			
Market access	0.16 ± 0.12	-0.15 ± 0.12	0.18 ± 0.17
****			
Steepness	-0.08 ± 0.12	-0.14 ± 0.16	-2.21 ± 0.48
****			
Population Density 1990	0.56 ± 0.20	-0.56 ± 0.25	1.43 ± 0.56
****			
Percent Agriculture 1992	0.05 ± 0.34	2.51 ± 0.46	1.33 ± 0.18
****			
GDP 1992	-0.10 ± 0.10	-0.42 ± 0.13	-0.39 ± 0.18
****			
Frontierness	0.78 ± 0.34	-0.98 ± 0.35	1.20 ± 0.53
****			
Frontierness * Percent Agriculture 1992	-0.10 ± 0.46	-2.55 ± 0.44	-0.07 ± 0.39
****			
Bioclimatic suitability * Market Access	-0.07 ± 0.11	0.09 ± 0.12	0.06 ± 0.16
****			
Steepness * Market Access	-0.03 ± 0.11	0.09 ± 0.11	-0.24 ± 0.22
****			
Bioclimatic suitability * Population Density 1990	-0.10 ± 0.13	-0.04 ± 0.15	-0.33 ± 0.16
****			
Steepness * Population Density 1990	-0.00 ± 0.14	0.01 ± 0.17	-0.15 ± 0.29
****			
Market Access * Percent Agriculture 1992	-0.09 ± 0.10	-0.34 ± 0.12	-0.40 ± 0.17
****			
Steepnes * Percent Agriculture 1992	0.23 ± 0.15	0.62 ± 0.17	0.02 ± 0.37
****			
Bioclimatic suitability * Percent Agriculture 1992	-0.09 ± 0.10	-0.21 ± 0.11	-0.24 ± 0.18
****			
Population Density 1992 * Percent Agriculture 1992	-0.31 ± 0.23	-1.28 ± 0.24	-0.11 ± 0.44
****			
Market Access * GDP 1992	-0.04 ± 0.10	0.06 ± 0.12	-0.84 ± 0.24
****			
Steepness * GDP1992	0.08 ± 0.11	0.11 ± 0.13	0.01 ± 0.26
****			
Bioclimatic suitability * GDP1992	-0.02 ± 0.10	-0.26 ± 0.12	-0.13 ± 0.13
****			
Population Density 1990 * GDP1992	-0.00 ± 0.13	0.18 ± 0.16	-0.18 ± 0.22
****			
McFadden Pseudo R2	0.20 ± 0.02	0.31 ± 0.02	0.41 ± 0.03

Table S5– Comparison of the overall model explanatory power (pseudo R^2) of the main cropland classification ('main') for the 'extent' models with cropland that includes mosaic land cover classes ('mosaic'). See Supplementary Methods for Details.

Model type	Cropland Threshold	Main Analysis	Mosaic
Extent	0.005	0.32	0.34
Extent	0.1	0.30	0.32
Extent	0.5	0.33	0.33

Table S6 – Comparisons of the overall model predictive power (pseudo R^2) main cropland classification ('main') for the 'expansion' models with cropland that includes mosaic land cover classes ('mosaic'), and cropland expansion up to 2013 ('2013'), rather than up to 2015. See Supplementary Methods for Details.

		Model 2 (Cropland + null)			Model 3 (Cropland + null + frontierness)		
Expansion	Cropland Threshold	Main Analysis	Mosaic	2013	Main Analysis	Mosaic	2013
Expansion	0.005	0.12	0.11	0.12	0.20	0.20	0.20
Expansion	0.1	0.23	0.16	0.23	0.31	0.32	0.32
Expansion	0.5	0.41	0.30	0.41	0.41	0.41	0.41

Table S7– Comparison of the overall model explanatory power (pseudo R^2) for 'extent' models for 2001, and predictive power (pseudo R^2) of the 'expansion' models (between 2001-2015) based on cropland as quantified by the ESA CCI data ('ESA CCI') and MODIS 6 data ('MODIS'). See Supplementary Methods for Details.

	Extent Model (Model 1)		Model 2 (Cropland + null)		Model 3 (Cropland + null + frontierness)	
Cropland Threshold	ESA CCI	MODIS	ESA CCI	MODIS	ESA CCI	MODIS
0.005	0.29	0.29	0.09	0.35	0.16	0.53
0.1	0.28	0.29	0.23	0.42	0.29	0.57

Table S8 – Proportional overlap of expansion of cropland between 1992 and 2015, and socio-economic variables of interest. Ratios > 1 indicate over-representation; ratios < 1 indicate under-representation (more or less overlap of cropland and a given variable than would be expected if both are equally common across the land area they cover)[15]. Details on the variables are in the Suppplementary Methods.

Variable	Expansion of cropland 1992-2015		
	0.5% crop	10% crop	50% crop
CurtisCommercial Agriculture	1.87	2.62	3.09
Curtis_Subsistence Agriculture	1.18	1.27	1.08
Largest quartile of field sizes	1.33	1.72	2.29
Smallest quartile of field sizes	1.35	1.49	1.28
GDP Changes 1992_2015	0.99	1.01	0.87
HDI Changes 1992_2015	0.99	1.04	1.01
Change in population density 1990_2015	0.97	0.97	0.94
Irrigated areas	1.13	1.36	0.85
Oil palm cultivation	1.79	1.86	1.32
Soy cultivation	1.29	2.18	3.32

Table S9: Proportional overlap of shifts from forest to short vegetation (1982-1992 and 1992-2015) and large positive deviations from the global 'null' model of cropland extent in 1992 (Methods; Null Model). 'Positive Deviances' of 2 or more (or 1 or more) standard deviations are shown. Ratios > 1 indicate over-representation; ratios < 1 indicate under-representation (more or less overlap of cropland and a given deviation threshold than would be expected if both are equally common across the land area they cover)[15]. As the positive deviance analysis was conducted using separate Null Models for different percent cropland binary cut-offs (>0.5%, >10%; >50%), separate overlaps were conducted for each thresholds. The shifts from forest to short vegetation are measured using AVHRR data following [9]; see Supplementary Methods for details.

	**Forest to short veg 1982-1992**			**Forest to short veg 1992-2015**		
	0.5% crop	10% crop	50% crop	0.5% crop	10% crop	50% crop
Positive Deviance 2 SD	3.84	3.12	1.84	3.48	1.29	1.11
Positive Deviance 1 SD	1.79	1.43	0.72	1.51	0.85	0.26

Supplementary References

1. Kummu, M., Taka, M., and Guillaume, J.H.A. (2018). Gridded global datasets for Gross Domestic Product and Human Development Index over 1990-2015. Sci. Data *5*, 1–15.
2. Gridded Population of the World, Version 3 (GPWv3): Population Density Grid, Future Estimates (2005).
3. Lesiv, M., Laso Bayas, J.C., See, L., Duerauer, M., Dahlia, D., Durando, N., Hazarika, R., Kumar Sahariah, P., Vakolyuk, M., Blyshchyk, V., et al. (2019). Estimating the global distribution of field size using crowdsourcing. Glob. Chang. Biol. *25*, 174–186.
4. FAO-AQUASTAT (2016). Food Agric. Organ. United Nations (FAO). AQUASTAT Database.

http://www.fao.org/nr/water/aquastat/data/query/index.html?lang=en.
5. Curtis, P.G., Slay, C.M., Harris, N.L., Tyukavina, A., and Hansen, M.C. (2018). Classifying drivers of global forest loss. Science (80-.). *361*, 1108–1111.
6. You, L., Wood, S., Wood-Sichra, U., and Wu, W. (2014). Generating global crop distribution maps: From census to grid. Agric. Syst. *127*, 53–60.
7. le Polain de Waroux, Y., Garrett, R.D., Graesser, J., Nolte, C., White, C., and Lambin, E.F. (2019). The Restructuring of South American Soy and Beef Production and Trade Under Changing Environmental Regulations. World Dev. *121*, 188–202.
8. le Polain de Waroux, Y., Baumann, M., Gasparri, N.I., Gavier-Pizarro, G., Godar, J., Kuemmerle, T., Müller, R., Vázquez, F., Volante, J.N., and Meyfroidt, P. (2018). Rents, Actors, and the Expansion of Commodity Frontiers in the Gran Chaco. Ann. Am. Assoc. Geogr. *108*, 204–225.
9. Song, X.P., Hansen, M.C., Stehman, S. V., Potapov, P. V., Tyukavina, A., Vermote, E.F., and Townshend, J.R. (2018). Global land change from 1982 to 2016. Nature *560*, 639–643.
10. Zalles, V., Hansen, M.C., Potapov, P. V., Stehman, S. V., Tyukavina, A., Pickens, A., Song, X.P., Adusei, B., Okpa, C., Aguilar, R., et al. (2019). Near doubling of Brazil's intensive row crop area since 2000. Proc. Natl. Acad. Sci. U. S. A. *116*, 428–435.
11. Pérez-Hoyos, A., Rembold, F., Kerdiles, H., and Gallego, J. (2017). Comparison of global land cover datasets for cropland monitoring. Remote Sens. *9*.
12. Sulla-Menashe, D., and Friedl, M.A. (2018). User Guide to Collection 6 MODIS Land Cover (MCD12Q1 and MCD12C1) Product. 1–18.
13. Bingham, H.C., Deguignet, M., Lewis, E., Stewart, J., Juffe-Bignoli, D., MacSharry, B., Milam, A., and Kingston, N. (2019). User Manual for the World Database on Protected Areas and world database on other effective area-based conservation measures: 1.6.
14. Doxsey-Whitfield, E., MacManus, K., Adamo, S.B., Pistolesi, L., Squires, J., Borkovska, O., and Baptista, S.R. (2015). Taking Advantage of the Improved Availability of Census Data: A First Look at the Gridded Population of the World, Version 4. Pap. Appl. Geogr. *1*, 226–234.
15. Eigenbrod, F., Anderson, B.J., Armsworth, P.R., Heinemeyer, A., Jackson, S.F., Parnell, M., Thomas, C.D., and Gaston, K.J. (2009). Ecosystem service benefits of contrasting conservation strategies in a human-dominated region. Proc. R. Soc. B Biol. Sci. *276*, 2903–2911.
